# Umbilical outpouchings in Danish piglets and weaners: prevalence and clinical characteristics—a cross-sectional study at herd level

**DOI:** 10.1186/s40813-023-00352-8

**Published:** 2024-01-13

**Authors:** Marie-Louise Hansen, Tina Birk Jensen, Charlotte Sonne Kristensen, Inge Larsen, Ken Steen Pedersen

**Affiliations:** 1 Department of Veterinary and Animal Sciences, Faculty of Health and Medical Sciences, University of Copenhagen, Grønnegårdsvej 2, 1870 Frederiksberg C, Denmark; 2 SEGES Innovation, Agro Food Park 15, 8200 Aarhus, Denmark; 3 Ø-Vet A/S, Køberupvej 33, 4700 Næstved, Denmark

**Keywords:** Umbilical outpouching, Pig, Hernia, Piglet, Weaner, Sustainability

## Abstract

**Background:**

Umbilical outpouchings (UO) in pigs present a welfare concern because of ulceration risk and complications. Danish legislation requires pigs with larger UOs to be housed in sick pens with soft bedding, and some UO pigs might not be suited for transport. Because of this, many UO pigs are euthanized, adding to the costs of pig production. The true prevalence of UO is unknown as no scientific reports with randomly sampled herds exist. This study aimed to estimate the prevalence of UO in Danish piglets and weaners and describe their clinical characteristics: size, texture, reducibility, and occurrence of ulcers. Lastly, risk factors for the occurrence of ulcers on UOs were investigated.

**Results:**

A cross-sectional study was conducted in 30 Danish conventional herds, with at least 800 weaned pigs and 200 sows. The herds were selected randomly from the Danish Husbandry Register and visited once between September 2020 and May 2021. Piglets were examined during their last week in the farrowing unit, and weaners were examined between weeks three and eight after weaning. The abdominal area was palpated on all pigs, and all irregularities were recorded; the results presented are umbilical outpouchings measuring at least 2 × 2 cm. The within-herd prevalence of piglets with UO averaged 4.2% with a range from 0.8 to 13.6% between herds. The within-herd prevalence of weaners with UO averaged 2.9%, ranging from 1.0 to 5.3% between herds. Approximately 80% of the UOs were classified as small or medium (< 7 cm piglets/ < 11cm weaners). Large outpouchings had significantly higher odds of ulcer occurrence (OR = 9.9, p < 0.001).

**Conclusion:**

UOs are common in Denmark, with a prevalence of 2.9% in weaners and an estimated annual production of 32 million Danish pigs almost a million pigs are affected yearly. Most of these pigs will have a small or medium UO. If the pigs have large UOs the odds of ulcer occurrence increase significantly. Numerous of these pigs are wasted, challenging sustainability and economy. UOs might also affect the welfare of the pigs. More research is therefore needed, especially in the prevention of UOs.

## Background

Umbilical outpouchings (UO) in pigs are a clinical condition that poses a challenge for the pigs as well as the producers [1]. All UOs were previously considered to be umbilical hernias, but Andersen et al. [2] found that slaughter pigs recorded with an umbilical hernia had different aetiologies: the most frequent diagnoses were cysts with haemorrhagic or serous fluid followed by hernias with intestinal content. The study also showed that all sorts of combinations between hernias, cysts, fibrotic tissue, abscesses, and paddle-formed proliferations exist [2]. Since the various disorders were typically not distinguishable based on clinical findings, the term "umbilical outpouching" was introduced as a replacement for umbilical hernia [3].

UOs are suspected to have a multifactorial background; Both genetic as well as infectious backgrounds have been suggested as hypotheses [4], as well as the handling of pigs might be relevant (e.g. how the piglets are lifted).

Pigs with UO need extra management; Danish legislation requires pigs with large UO to be stabled in sick pens with soft bedding, and the risk of UO pigs being unfit for transport is increased compared to pigs without UO [5]. Some of the UO pigs can be approved for transport if the herd veterinarian provides them with a transport fitness certificate and they are transported under special conditions, which adds costs for keeping UO pigs. Therefore, a high proportion of UO pigs are euthanized, contributing to increased mortality, a poorer economy, and reduced sustainability for pig production.

The true prevalence of UO in intensive pig production is unknown. Earlier studies report varying prevalences and comparisons between studies are difficult because the definitions of UO vary considerably. Searcy-Bernal and Gardner [4] examined 2958 pigs weekly and found a cumulative incidence of 1.5% with a definition including only hernias with a hernia ring of more than one cm. Mattson et al. [6] found a cumulative incidence of 8.3% including both hernias, abscesses, and other navel problems, in five Swedish herds stated to experience problems.

Yun et al. [7] found occurrences between 0.7 and 2.3% including both hernias and abscesses in 6451 pigs in one Finnish herd.

This study aimed to obtain knowledge about UOs in different Danish herds, build a foundation for benchmarking between herds, and add to an increasing understanding of the condition, which in the future can be used to generate new preventive interventions. A cross-sectional study was performed with three objectives:

The primary objective was to estimate the within- and between-herd prevalence of UOs in Danish piglets and weaner pigs.

The second objective was to describe the clinical characteristics of UOs such as size, texture, reducibility, and occurrence of ulcers.

The third objective was to identify risk factors for the occurrence of ulcers on UOs.

## Results

480 conventional herds fulfilled the inclusion criteria. From a randomised list of the latter, a total of 62 herds were contacted, and 30 herd owners agreed to participate. The sample size within each herd ranged from 115–530 for piglets and 448–853 for weaners. A total of 8052 piglets and 19,684 weaners were clinically examined. More than 90% (28/30) of the herds treated all piglets with antibiotics within 48 h postpartum in varying schemes. Of the two not using systematic antibiotics, one was in transition to becoming Danish Crown Pure Pork [8], whereas the other was a conventional herd.

**Fig. 1:**
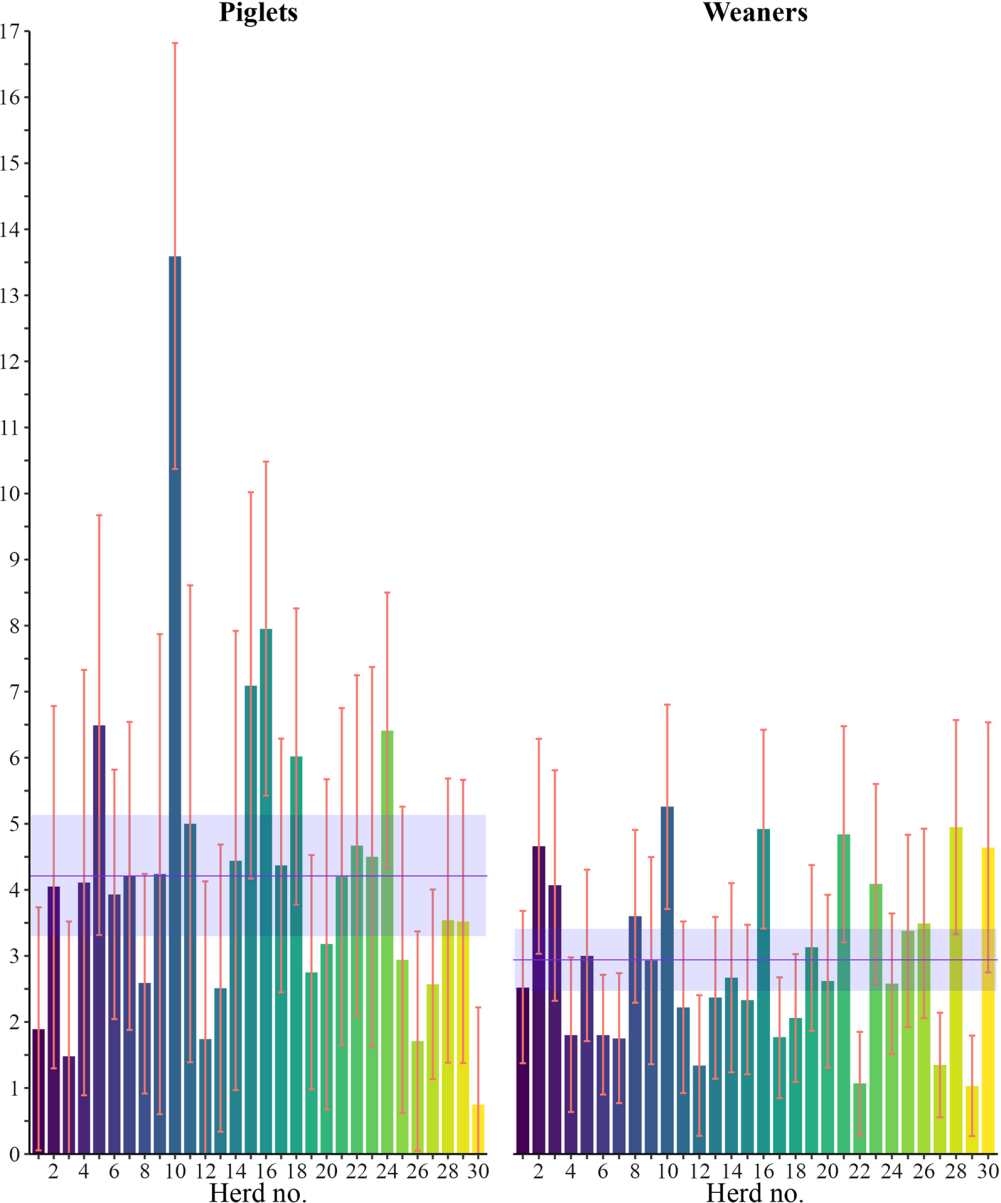
Prevalence of umbilical outpouchings including confidence intervals for each herd. The purple horizontal line is the average within-herd prevalence and confidence interval

Figure 1 shows the prevalence for each herd including their confidence intervals for both age groups. There were no correlations between the levels of outpouchings in piglets and weaners in individual herds.

The average within-herd prevalence^1^ of piglets with UO was 4.2% CI [3.3–5.1] ranging from 0.8 to 13.6% between herds, with a median of 4.1%.

The average within-herd prevalence of weaners with UO was 2.9% CI [2.5–3.4] ranging from 1.0 to 5.3% between herds, with a median of 2.7%.

Only seven herds had sick pens and therefore the possibility to move UO pigs to the sick pens (herds 17, 18, 19, 21, 22, 23, 27 & 29), thereby maybe introducing a false lower prevalence. Comparing the seven herds with sick pens to the 23 herds without sick pens revealed a significantly lower prevalence of total UO in herds with sick pens (2.1 vs 3.2, p = 0.035), with the same distribution of small, medium, and large outpouchings as the herds without sick pens (Table 1).

**Table 1:** UO % in piglets and weaners including confidence interval, minimum and maximum values

%	Piglets	Weaners		
	All herds	All herds	Herds without sick pens	Herds with sick pens
N herds N pigs N UO pigs	30 8052 380	30 19,684 579	23 14,515 473	7 5169 106
Total UO [CI] Min–max	4.2 [3.3–5.1] 0.8–13.6	2.9 [2.5–3.4] 1.0–5.3	3.2 [2.7–3.7] 1.3–5.3	2.1 [1.0–3.1] 1.0–4.1
Small UO* [CI] Min–max	63.6 [56.2–71.0] 25–100	56.9 [51.3–62.6] 25–92.9	56.8 [51.2–62.4] 25–84.6	57.5 [37.3–77.7] 34.8–92.9
Medium UO* [CI] Min–max	20.7 [14.9–26.6] 0–50	24.2 [20.0–28.4] 0–42.9	24.9 [20.4–29.4] 0–41.67	22 [8.9–35] 0–42.9
Large UO* [CI] Min–max	15.7 [10.7–20.6] 0–40	18.8 [14.1–23.6] 0–43.8	18.3 [13.5–23.1] 0–43.75	20.5 [3.5–37.6] 0–42.9

Distribution of outpouchings in size categories across the four groups; Piglets, weaners, herds with sick pens, and herds without sick pens

*Percentage of the total number of UO

For all groups the small outpouchings were dominant, and the large outpouchings were the fewest.

Approximately 60% of the pigs with outpouchings were females for both piglets and weaners, data are shown in Table 2.

**Table 2:** Number of UO pigs and the total number of examined piglets and weaners

Sex	Piglets N (%)		Weaners N (%)	
	UO yes	Pigs total	UO yes	Pigs total
Male	153 (40.3)	3976 (49.4)	231 (39.9)	677(3.4)
Female	226 (59.5)	3944 (49)	347 (59.9)	645 (3.3)
NA	1 (0.3)	132 (1.6)	1 (0.2)	18,362 (93.3) *
Total	380 (100)	8052	579 (100)	19,684

*Not all weaners have sex registered due to the study design

*NA*: Not available

Table 3 shows the prevalence of clinical characteristics of the outpouchings found in piglets and weaners. For all groups, the majority of the UOs were nonreducible and soft in texture. Less than one percent of the piglets with UOs had ulcers, whereas more than 10 percent of the weaners had ulcers on their outpouchings.

**Table 3:** Within herd prevalence of clinical characteristics of the outpouchings per age groups and herds with and without sick pens

		Piglets	Weaners			
		All herds	All herds	Herds without sick pens	Herds with sick pens	Pigs in sick pens
	N herds N pigs N UO pigs N ulcers	30 8052 380 4	30 19,684 579 69	23 14,515 473 60	7 5169 106 9	7 554 176 30
Reducible	Yes, % [CI] Min–Max	32.3 [24.2–40.4] 0–70.6	21 [15.9–26.1] 0–50	20.0 [13.8–26.2] 0–50	24.2 [13.6–34.8] 11.1–42.9	18.1 [5.0–31.2] 6.2–47.1
	Partly, % [CI] Min–Max	10.7 [5.2–16.1] 0–50	13.3 [9.6–17.1] 0–35.7	13.4 [9.0–17.8] 0–35.7	13.2 [3.8–22.7] 0–28.6	19.2 [13.4–25.0] 12.5–29.4
	No, % [CI] Min–Max	57 [48.0–66.0] 12.5–100	65.7 [60.0–71.7] 28.6–100	66.7 [60.2–73.2] 41.7–100	62.6 [44.6–80.6] 28.6–81.8	62.7 [44.8–80.7] 23.5–81.2
Texture	Soft, % [CI] Min–Max	56.5 [46.3–66.6] 0–100	48 [41.8–54.2] 9.1–85.7	46.5 [40.5–52.5] 9.1–66.7	53.1 [30.8–75.3] 18.2–85.7	56.8 [36.3–77.4] 25–87.5
	Mix, % [CI] Min–Max	1.7 [0.3–3.0] 0–13.3	7.4 [4.8–10.0] 0–27.3	7.11 [4.0–10.3] 0–27.3	8.5 [2.9–14.0] 0–14.3	9.4 [0–22.2] 0–37.5
	Hard, % [CI] Min–Max	40.7 [30.4–51.0] 0–100	43.5 [36.6–50.5] 0–81.8	45.1 [38.1–52.1] 22.7–81.8	38.5 [14.1–62.8] 0–72.7	33.8 [15.9–51.8] 11.8–66.7
	NA, % [CI] Min–Max	1.2 [0–2.9] 0–25	1 [0–2.3] 0–16.7	1.3 [0–3] 0–16.7	0	0
Ulcer	Yes, % [CI] Min–Max	0.7 [0–1.5] 0–10.5	12.5 [7.9–17.2] 0–57.1	12.6 [8.4–16.8] 0–33.3	12.4 [0–30.9] 0–57.1	15.2 [7.1–23.4] 0–25
	No, % [CI] Min–Max	98.9 [97.9–99.9] 89.5–100	86.7 [82.0–91.3] 42–100	87.0 [82.7–91.2] 66.7–100	85.8 [67.6–100] 42.9–100	84.6 [76.4–92.8] 75–100
	NA, % [CI] Min–Max	0.4 [0–1.0] 0–8.3	0.8 [0.1–1.5] 0–7.1	0.48 [0–1.1] 0–5.1	1.8 [0–4.8] 0–7.1	0.2 [0–0.5] 0–1.1
Ulcer size	Small, % [CI] Min–Max	*	22.7 [8.3–37.1] 0–100	23.4 [7.8–39] 0–100	20 [0–75.5] 0–100	33.9 [0–78.2] 0–100
	Medium, % [CI] Min–Max	*	54.3 [37.7–70.8] 0–100	59.3 [41.8–76.8] 0–100	35 [0–95.5] 0–100	57 [15.5–98.5] 0–100
	Large, % [CI] Min–Max	*	19.2 [6.2–32.3] 0–33.3	12.5 [2.8–22] 0–50	45 [0–100] 0–100	3.5 [0–12.5] 0–33.3
	NA, % [CI] Min–Max	*	3.8 [0–8.2] 0–33.3	4.8 [0–10.4] 0–33.3	0	5.6 [0–19.8] 0–33

*Not enough data available

When focusing on weaners with UO, size, reducibility, and texture were considered risk factors for the occurrence of ulcers.

Table 4 shows the results from the univariable and multivariable analyses of the risk factors with the outcome ulcer. Based on those results the odds of developing an ulcer on the UO was significantly higher when the UO was classified as medium (OR = 3.8, p < 0.001) or large (OR = 9.9, p < 0.001) compared to small UOs. In the multivariable analysis, the texture of the UO was not statistically associated with the development of ulcers (p = 0.087), whereas weaners with non-reducible or partly reducible UOs had significantly higher odds (OR = 2.4, p = 0.017) of developing an ulcer compared to weaners with a reducible UO.

**Table 4:** Univariable and multivariable analysis—ORs for variables considered risk factors for ulcer occurrence

Variable	Univariable			Multivariable		
	Level	OR (95% CI)	P value	Level	OR (95% CI)	P value
Size category UO	Small	1		Small	1	
	Medium	3.8 (2.1–7.2)	< 0.001	Medium	3.8 (2.0–7.2)	< 0.001
	Large	9.7 (5.6–17.7)	< 0.001	Large	9.9 (5.6–18.4)	< 0.001
Reducibility	Yes	1		Yes	1	
	Partly	2.7 (1.2–6.3)	0.0143	Partly/ no	2.4 (1.2–5.2)	0.017
	No	1.9 (1–4.1)	0.0628	–	–	
Texture UO	Soft	1		Soft	1	
	Mix	2 (0.9–4.1	0.0826	Mix/ hard	0.8 (0.3–1.7)	0.087
	Hard	2.2 (1.4–3.6)	< 0.001	–	–	

## Discussion

This study provided good estimates for the prevalence of UO within Danish herds; it does not, however, tell the true prevalence of UO, since management procedures in the herds affect the observed prevalence.

An example of this is the use of sick pens (which are mandatory by law in Denmark), which lowers the observed prevalence in our random sample. Many herds, including herds participating in this study, routinely euthanize pigs with UO. The study’s voluntary participation could favour herds more affected by outpouchings or make herds with problems more likely to decline to take part, which is another bias.

The study confirmed our prior expectation of differences between herds and a general level of approximately three percent UOs in the weaners, it also showed a higher level of UOs in the piglets. Especially in the farrowing unit, the prevalence varied between the herds. We cannot, however, tell what caused the differences, a possible explanation is different weaning ages between the herds and as such more or less healed/ inflamed umbilici and concurrent swellings. We know from other studies that UOs might disappear/ appear as the pigs grow [6, 9, 10] thereby affecting the observed prevalence.

The variation in the prevalence of UOs in the weaners is more easily explained and strongly relates to management procedures and conditions in the stable market.

If the herds are dependent on selling all their weaners they will probably euthanize more UO pigs, because they will have less tolerance for UO pigs, compared to herds who can sell UO pigs as roaster pigs or keep UO pigs in sick pens or finisher stables until slaughter. The relationship between the listing price of pig meat and the cost of feeding the animals is also an important factor when farmers decide whether to keep UO pigs or not.

The main reason behind fewer pigs showing outpouchings in this study compared to previous Danish studies [10, 11] lies in the use of different definitions of umbilical outpouchings.

Larsen et al. [10] examined pigs in two herds not using systematic antibiotics at birth and found an incidence of UOs of 9.5% including every finding of a firm protrusion or a rounded protrusion at the umbilicus. More than half of the UOs found at 5 weeks of age had disappeared when the pigs were 12 weeks old.

Hovmand-Hansen et al. [9, 11] found an incidence of 8% UO pigs in two commercial herds with a history of UO problems., and spontaneous regression was seen in 14% of the UO pigs. A UO was defined as a protrusion of more than 0.5 cm.

This study focused on what we consider clinically relevant outpouchings, hence the introduction of a cut-off value for the size of UO. Petersen et al. [12] used a similar definition and found less than one percent of pigs with “a visible bulge at the umbilicus” when examining finisher pigs, not providing data from the sick pens, and knowing that many pigs with UO might have been euthanized before they reached the finisher unit.

The apparent higher occurrence of UO among female pigs has also been found in other studies [10, 11]. The reasons for this are unknown.

Even though herds with sick pens did have a lower occurrence of pigs with UOs in their ordinary pens, they still had the same distribution of small, medium, and large UOs. One would expect that they would have had at least fewer large outpouchings. This probably reflects the fact that UOs are quite hard to spot and when they are found it is often by chance.

The risk factor analysis for ulcers agrees with other studies [9]. Hovmand-Hansen et al. [9, 11] also found that large outpouchings were associated with higher odds for the occurrence of ulcers and that reducible outpouchings had lower odds, even though the size definitions of outpouchings were not the same as the ones in this study.

This study demonstrated that there were very few pigs with large ulcers in the sick pens, which likely reflects the fact that pigs in sick pens are more closely monitored and perhaps that pigs with large ulcers are deemed unlikely to heal and therefore euthanized when they are found, more than it reflects a healing effect of the sick pens. Euthanasia is often the most reasonable cause of action since a large ulcer makes the pig unfit for transport.

## Conclusions

UOs are common in Denmark, with a prevalence of 2.9% in weaners and an estimated annual production of 32 million Danish pigs [13] almost a a million pigs are affected yearly. Most of these pigs will have a small or medium UO. If the pigs have large outpouchings the odds of ulcer occurrence increase significantly. Numerous of these pigs are wasted, challenging sustainability and economy. Also, UO's possible effects on the welfare of the pigs need to be considered. More research is therefore needed, especially in the prevention of UOs.

Another possibility is exploring the utilisation of mobile slaughter solutions. Processing the pigs directly at the farm would spare them the stress of transport, and minimize the number of wasted pigs, thereby making pig production more sustainable and humane.

## Methods

### Study design

A cross-sectional study was performed in 30 conventional herds visited once between September 2020 and May 2021. Piglets were examined the last week before weaning and weaners were examined between weeks three and eight after weaning. Sampling was performed at pen level by random selection of pens, and all pigs housed in sick pens were examined as a separate group (e.g. they were not part of the random sampling in the herd). The abdominal area was palpated on all selected pigs and all irregularities were recorded. UOs measuring at least 2 × 2 cm were reported as UOs in this study.

### Sample size

Herd was the primary study unit of interest. Based on project-budget and logistic considerations it was possible to collect data from thirty herds. Thirty herds were considered sufficient for obtaining a representative sample of the Danish conventional pig population and to obtain a valid estimate of the average within-herd prevalence of pigs with UOs.

To estimate the within-herd prevalence Eq. 1 [14] was used to calculate the sample size.

1 $${{\text{N}}}_{{\text{pigs}}}= \frac{{{\text{Z}}}_{1-\frac{\mathrm{\alpha }}{2}}^{2} \times \mathrm{ p }\left(1-{\text{p}}\right)}{{{\text{L}}}^{2}}$$

Calculation of sample size to estimate a proportion.

Based on the literature a presumed UO prevalence (P) was set to 2.5% and maximum allowable error (L) was set to 1%. With a 95% confidence level, the resulting sample size was 937 pigs in each age group in each herd. The sample size was then adjusted for herd size using Eq. 2 [14]. For piglets, n_population_ was the number of weaned pigs per week in the specific herd, and for weaners, n_population_ was the number of (weaned pigs/week) times six weeks (weeks 3–8 post-weaning). Thus, the sample sizes for a herd weaning 500 pigs a week were 327 piglets and 714 weaners.

2 $${{\text{N}}}_{{\text{adjusted}}}=\frac{1}{\frac{1}{{{\text{n}}}_{{\text{pigs}}}}+\frac{1}{{{\text{n}}}_{{\text{population}}}}}$$

Calculation of adjusted sample size.

### Selection of herds and pigs

In July 2020 a list of pig herds was retrieved from the Danish Husbandry Register (CHR database). Inclusion criteria for herds were at least 200 sows and 800 weaned pigs registered on the same CHR number, and being within a three-hour drive from Copenhagen. Secondly, herds should use either Danbred or Danish Genetics and keep pigs for the entire nursery period. Pigs had to be crossbreds between Landrace/ Yorkshire/Duroc.

The homepage https://www.randomizer.org/ was used to find 30 random herds, using the “math.random” method from the JavaScript programming language [15].

Herds appointed by the research randomizer were contacted by phone and asked to participate if they fulfilled the inclusion criteria. New random herds were drawn if herds did not fulfil the inclusion criteria, contact was not established, or herds declined to participate.

The study population was piglets within one week before weaning and weaned pigs between three and eight weeks after weaning. Pigs were selected at pen level and all pigs in selected pens were subjected to clinical examination. Every *n*th pen was examined based on the required adjusted sample size, the number of pens with weaners at the required age, and the number of pigs in each pen.$\frac{No\,of\,pens\,with\,weaners\,of\,required\,age}{{Adjusted\,sample\,size\,based\,on\,herd\,size}/{No\,of\,pigs\,in\,each\,pens}}=every\,{n}^{th}\,pen$^2^

To ensure equal age distribution for the weaners, the number of included pens was divided equally between all weaner rooms with weaners at the right age.

All pigs housed in sick pens were examined as a separate group, and not as part of the random sample.

### Clinical examination

The piglets were lifted by technicians and palpated by one veterinarian. If there was any confusion or uncertainty about findings, findings were confirmed visually.

The weaners were screened by trained technicians who palpated the abdominal area of all pigs. Every pig with an abnormality, bulge, or uncertainty was spray-marked by the technicians; as a result, only weaners with suspected outpouchings were examined by the vet and had sex recorded. Marked pigs were fixated with a herding board against a corner of the pen and examined standing. One veterinarian examined all the pigs. For pigs with outpouchings the height and width in cm were registered, as well as reducibility (yes, partly, no), ulcers (yes, no), ulcer size (length x width cm), and texture (soft, mix, hard). The outpouchings and ulcers were categorised into three categories based on the sum of the height and width of the UO,^3^ and the length and width of the ulcer, as shown in Table 5.

**Table 5:** Categorisation of UO size and ulcers into categories—small, medium & large

	Umbilical outpouching category			Ulcer size category		
	Small	Medium	Large	Small	Medium	Large
Piglets	4 cm	5–6 cm	≥ 7 cm	2 cm	3–4 cm	≥ 5 cm
Weaners	4–7 cm	8–10 cm	≥ 11 cm	2–3 cm	4–7 cm	≥ 8 cm

The outpouchings are classified into one category based on the sum of their height and width in cm, the same applies to ulcer size where length and width are used

### Statistical analysis

The herd is the experimental unit for all analyses except for the analysis of ulcers where the experimental unit is individual pigs with UO. All data were analysed, and graphs were made, in Rstudio [16] using functions from the Tidyverse package [17]. Comparisons between herds with and without sick pens were made using the T.test following the Shapiro–Wilk normality test and the F.test for comparing variance. Linear regression was used to look for correlations between piglets and weaners in individual herds.

Risk factors for the occurrence of ulcers were first assessed by univariable analysis. Levels were reduced based on significant p-values and estimates before the multivariable model was built. For reducibility “partly” and “no” were combined because they had similar estimates and no significant differences, and the same applies to texture where “mix” and “hard” were combined. A p-value lower than 0.05 was considered significant.

## Data Availability

The datasets used and analysed during the current study are available from the corresponding author upon reasonable request.

## Footnotes

1 Calculated based on the herd means.

2 Example: $$\frac{20\,pens}{100\,pigs/ 25 pigs\,in\,each\,pen}=every\,{5}^{th}\,pen\,examined$$

3 Example: An UO in a weaner measuring 4 times 5 cm, sum = 9 size medium.
